# GSDME couples pyroptosis to lipid reprogramming

**DOI:** 10.1093/lifemeta/loag026

**Published:** 2026-09-03

**Authors:** Rui Kang, Daolin Tang

**Affiliations:** Department of Surgery, UT Southwestern Medical Center, Dallas, TX 75390, the United States; Department of Surgery, UT Southwestern Medical Center, Dallas, TX 75390, the United States

Regulated cell death is an integral part of the host response to viral infection [1, 2]. Although apoptosis has long been considered a relatively immunologically silent mechanism for eliminating infected cells, pyroptosis leads to membrane rupture and the release of inflammatory mediators and intracellular danger signals, including damage-associated molecular patterns (DAMPs) [3]. Gasdermin E (GSDME) occupies an unusual position between these two forms of cell death because cleavage by caspase-3 (CASP3), a canonical apoptotic caspase, converts GSDME into a pore-forming effector [4, 5]. This mechanism has established GSDME as an important determinant of how apoptotic signaling is translated into inflammatory cell death. What has been less clear is whether GSDME has functions beyond membrane permeabilization.

In a recent study published in *Cell*, Wang *et al.* [6] examined this question in the setting of wetland virus (WELV) infection. WELV is an emerging tick-borne orthonairovirus associated with human febrile illness [7]. The study identifies a pathogenic pathway in which viral RNA sensing activates CASP3-dependent GSDME cleavage in hepatocytes, while GSDME also promotes lipid accumulation through a direct effect on fatty acid synthase (FASN) (Fig. 1). These two activities converge on severe hepatic steatosis and lethal disease in mice.

**Figure 1:**
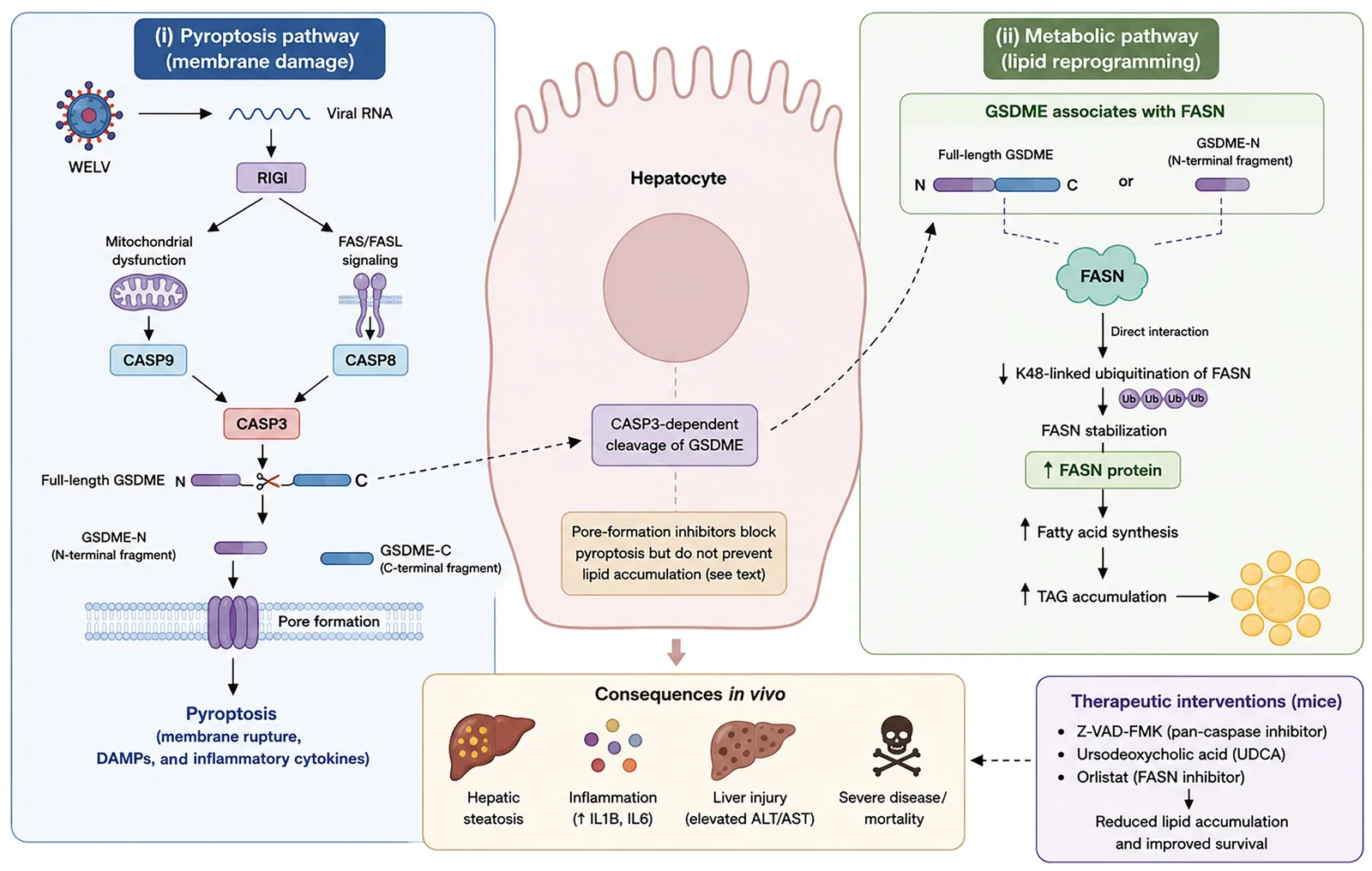
GSDME couples WELV-induced pyroptosis to hepatic lipid accumulation. WELV RNA sensing through RIGI activates both the CASP9-CASP3 and FAS/FASL-CASP8-CASP3 pathways. CASP3 cleaves GSDME to generate GSDME-N, which forms membrane pores and promotes pyroptotic cell death, leading to the release of DAMPs and inflammatory mediators. In parallel, both full-length GSDME and GSDME-N interact with FASN, reducing its K48-linked ubiquitination and proteasomal degradation. FASN stabilization promotes fatty acid synthesis and TAG accumulation, contributing to hepatic steatosis, inflammation, liver injury, and severe disease. Pharmacological inhibition of caspase signaling or FASN reduces lipid accumulation and improves survival in WELV-infected mice. CASP, caspase; DAMPs, damage-associated molecular patterns; FAS, Fas cell surface death receptor; FASL, FAS ligand; FASN, fatty acid synthase; GSDME, gasdermin E; GSDME-N, N-terminal fragment of GSDME; RIGI, retinoic acid-inducible gene I; TAG, triacylglycerol; WELV, wetland virus.

Clinical observations first prompted the authors to consider whether altered lipid metabolism might contribute to WELV pathogenesis. Among 27 patients analyzed, 10 showed evidence of liver injury, with elevated liver enzymes and reduced albumin. Levels of serum triacylglycerol (TAG), interleukin-1 beta (IL1B, also known as IL-1β), and IL6 were also increased in patients with liver injury and correlated with viral load. Three patients with persistent liver dysfunction and reduced hepatic attenuation on computed tomography subsequently died. Although the patient cohort was small, the association between hepatic injury, systemic inflammation, and altered TAG metabolism prompted the authors to examine whether these processes were mechanistically linked.

Using cultured cells and primary human hepatocyte-derived liver organoids, Wang *et al.* showed that WELV infection induced a form of cell death characterized by GSDME cleavage, membrane swelling, lactate dehydrogenase release, and inflammatory cytokine production. Depletion of either CASP3 or GSDME reduced these responses. Notably, the process did not depend on canonical inflammasome activation or CASP1, distinguishing it from the more familiar GSDMD-dependent form of pyroptosis.

The upstream signaling is more complex than a single linear pathway. WELV activates both mitochondrial and death receptor signaling. Mitochondrial dysfunction promotes CASP9 activation, whereas induction of Fas cell surface death receptor (FAS) and FAS ligand (FASL) engages CASP8. Both pathways converge on CASP3 and GSDME. Viral RNA appears to initiate much of this response through RNA sensor retinoic acid-inducible gene I (RIGI). Knockdown of *Rigi*, but not Toll-like receptor 7 (*Tlr7*), *Tlr8*, or interferon-induced with helicase C domain 1 (*Ifih1*, also known as *Mda5*), strongly impaired activation of CASP8, CASP9, and CASP3 and reduced subsequent GSDME-dependent pyroptosis. Thus, in this setting, an innate RNA-sensing pathway is coupled to both intrinsic and extrinsic apoptotic machinery, with GSDME providing a route by which CASP3 activation can progress to lytic inflammatory cell death.

The virus itself also appears to modulate this process. WELV nucleoprotein (NP) contains a CASP3 cleavage motif and interacts with activated CASP3. CASP3-dependent cleavage of NP is associated with reduced GSDME processing, suggesting that NP may compete with GSDME for CASP3. The authors interpret this as a negative-feedback mechanism that may restrain excessive pyroptosis during infection. This aspect of the study is particularly relevant to orthonairovirus biology, because related viral nucleoproteins also contain conserved caspase-cleavage motifs. Whether this mechanism primarily benefits viral replication, limits host tissue damage, or does both at different stages of infection remains to be determined.

The central advance of the study, however, lies in the connection between GSDME and lipid metabolism. WELV-infected mice developed marked hepatomegaly, lipid-droplet accumulation, elevated hepatic and circulating TAG, and extensive remodeling of the liver lipidome. These changes were accompanied by increased FASN protein despite reduced *Fasn* transcript abundance, pointing to a post-transcriptional mechanism.

Genetic deletion of *Gsdme* provided further support for this model. WELV infection was uniformly lethal in wild-type mice under the experimental conditions, whereas *Gsdme*-deficient mice survived despite comparable viral loads. Loss of *Gsdme* also reduced liver injury and inflammatory cytokines, as well as hepatic TAG and free fatty acids, and was associated with lower FASN abundance. These findings indicate that GSDME contributes to disease severity independently of a major effect on viral replication and place host metabolic injury downstream of the cell-death pathway.

Mechanistically, the authors found that GSDME physically associates with FASN. Both full-length GSDME and its N-terminal fragment interact with FASN, whereas the C-terminal fragment does not. Mutational analysis further implicated glutamate‑74 (E74) and aspartate‑76 (D76) in the interaction. More importantly, GSDME reduced lysine‑48 (K48)-linked ubiquitination of FASN, thereby limiting its proteasomal degradation. WELV infection simi­larly decreased K48-linked FASN ubiquitination, an effect that was lost in *Gsdme*-deficient cells. The resulting increase in FASN protein provides a mechanistic link to increased fatty acid availability and subsequent TAG accumulation in infected liver.

This observation changes how the role of GSDME in this study should be viewed. GSDME is not simply upstream of metabolic dysfunction because pyroptotic cells become damaged. Instead, it appears to act directly on a metabolic enzyme. The distinction is supported by experiments using inhibitors of GSDME pore formation. These agents reduced pyroptotic cell death but did not substantially prevent WELV-induced lipid-droplet accumulation. In contrast, loss of *Gsdme* itself strongly reduced lipid accumulation. The metabolic function of GSDME can therefore be at least partly separated from its membrane-lytic activity.

Previous studies have established close links between lipid metabolism and gasdermin-dependent pyroptosis. For example, lipid peroxidation promotes CASP11-GSDMD-mediated pyroptosis during polymicrobial sepsis, whereas glutathione peroxidase 4 restrains this process by limiting lipid peroxidation [8]. GSDMD-dependent pyroptosis has also been implicated in metabolic liver disease, including steatohepatitis [9]. The study by Wang *et al.* adds an interesting dimension to this relationship. Rather than acting solely as an executor of inflammatory cell death, GSDME directly influences lipid metabolism by stabilizing FASN [6]. Thus, the interaction between pyroptosis and lipid metabolism may operate in both directions: lipid metabolic states can determine the susceptibility to pyroptotic cell death, while gasdermin activation may, in turn, reprogram cellular lipid metabolism.

The therapeutic experiments are consistent with this model. Inhibition of the CASP3-GSDME arm reduced both inflammatory injury and lipid accumulation. The pan-caspase inhibitor Z-VAD-FMK suppressed GSDME cleavage, reduced FASN expression and hepatic steatosis, and substantially improved survival. Ursodeoxycholic acid, which inhibited CASP3 activation in this model, produced similar effects. Targeting the metabolic arm with the FASN inhibitor orlistat also reduced hepatic lipid accumulation and improved survival. Although the specificity and translational relevance of these interventions remain to be established, together, these results support the idea that inflammatory cell death and metabolic reprogramming are functionally connected components of the same pathogenic process.

There are, nevertheless, several issues that will require further investigation. How GSDME suppresses K48-linked ubiquitination of FASN remains unclear. It may alter the recruitment or activity of components of the FASN ubiquitination machinery, a possibi­lity that will require direct testing. Defining this mechanism will be important for determining whether the GSDME-FASN interaction represents a broader regulatory principle.

The lipid phenotype may also extend beyond FASN-dependent fatty acid synthesis. Lipidomic analysis showed substantial changes in phospholipids and sphingolipids, including accumulation of several atypical sphingolipid species. The functional contribution of these changes remains unresolved, as acknowledged by the authors. It is therefore possible that FASN stabilization is one component of a broader metabolic response to WELV infection rather than the sole cause of hepatic steatosis.

A second limitation concerns human disease. The study detected increased circulating CASP3 and GSDME in patients with WELV-associated liver injury, but direct evidence for GSDME cleavage and FASN stabilization in infected human hepatocytes *in vivo* is still lacking. Larger patient cohorts will also be required to determine whether changes in circulating TAG or other lipid species track with disease progression and have prognostic value.

The work by Wang *et al.* therefore extends the biology of GSDME beyond its established role as an executor of pyroptosis. In WELV infection, GSDME participates in two related but mechanistically separable processes: CASP3-dependent membrane damage and regulation of FASN stability. Their convergence provides a plausible mechanism linking antiviral cell-death signaling to acute hepatic lipid accumulation. It will now be important to determine whether similar gasdermin-dependent metabolic functions ope­rate in other viral infections and, more generally, in inflammatory and metabolic liver disease. More broadly, a better understanding of the metabolic basis of cell death may reveal new opportunities for therapeutic intervention [10].

## References

[1] Jorgensen I , Rayamajhi M, Miao EA. Nat Rev Immunol 2017;17:151–64. 10.1038/nri.2016.147 PMC5328506 28138137

[2] Upton JW , Chan FK. Mol Cell 2014;54:273–80. 10.1016/j.molcel.2014.01.027 PMC4010939 24766891

[3] Chen R , Zou J, Liu J et al Mol Cell 2025;85:3874–89. 10.1016/j.molcel.2025.09.007 41106375

[4] Wang Y , Gao W, Shi X et al Nature 2017;547:99–103. 10.1038/nature22393 28459430

[5] Rogers C , Fernandes-Alnemri T, Mayes L et al Nat Commun 2017;8:14128. 10.1038/ncomms14128 PMC5216131 28045099

[6] Wang C , Zheng X, Zhang Y et al Cell 2026. Doi: 10.1016/j.cell.2026.07.011.

[7] Zhang XA , Ma YD, Zhang YF et al N Engl J Med 2024;391:821–31. 10.1056/NEJMoa2313722 39231344

[8] Kang R , Zeng L, Zhu S et al Cell Host Microbe 2018;24:97–108.e4. 10.1016/j.chom.2018.05.009 PMC6043361 29937272

[9] Xu B , Jiang M, Chu Y et al J Hepatol 2018;68:773–82. 10.1016/j.jhep.2017.11.040 29273476

[10] Liu J , Wang J, Kang R et al Cell Metab 2026;38:1315–33. 10.1016/j.cmet.2026.06.001 42349417

